# Supplementary material for: Millennial-to-orbital-scale subsurface ocean warming and Polynya formation off Dronning Maud Land during the last glacial
Source: Nat Commun. 2026 Mar 12;17:2440. doi: 10.1038/s41467-026-70498-w (PMC12988150; doi:10.1038/s41467-026-70498-w)
Supplement: Supplementary file 1 — Supplementary Information [file 41467_2026_70498_MOESM1_ESM.pdf]

Supplementary Information for "Millennial-to-orbital-scale subsurface ocean warming and Polynya formation off Dronning Maud Land during the last glacial"

Tainã M. L. Pinho[1,2*]; Dirk Nürnberg[3]; A. Nele Meckler[4]; Gesine Mollenhauer[1,2,5]; Juliane Müller[1,5], Gerrit Lohmann[1,5]; Lester Lembke-Jene[1]; Salma Hidayat[2], Vincent Rigalleau[1] Frank Lamy[1], Ralf Tiedemann[1]

[1]Alfred Wegener Institute for Polar and Marine Research, Bremerhaven, Germany.

[2]University of Bremen, Faculty of Geosciences, Bremen, Germany.

[3]GEOMAR Helmholtz Centre for Ocean Research Kiel, Kiel, Germany.

[4]Bjerknes Centre for Climate Research and Department of Earth Science, University of Bergen, Bergen, Norway.

[5] University of Bremen, MARUM, Bremen, Germany.

*Corresponding author

e-mail: taina.pinho@awi.de

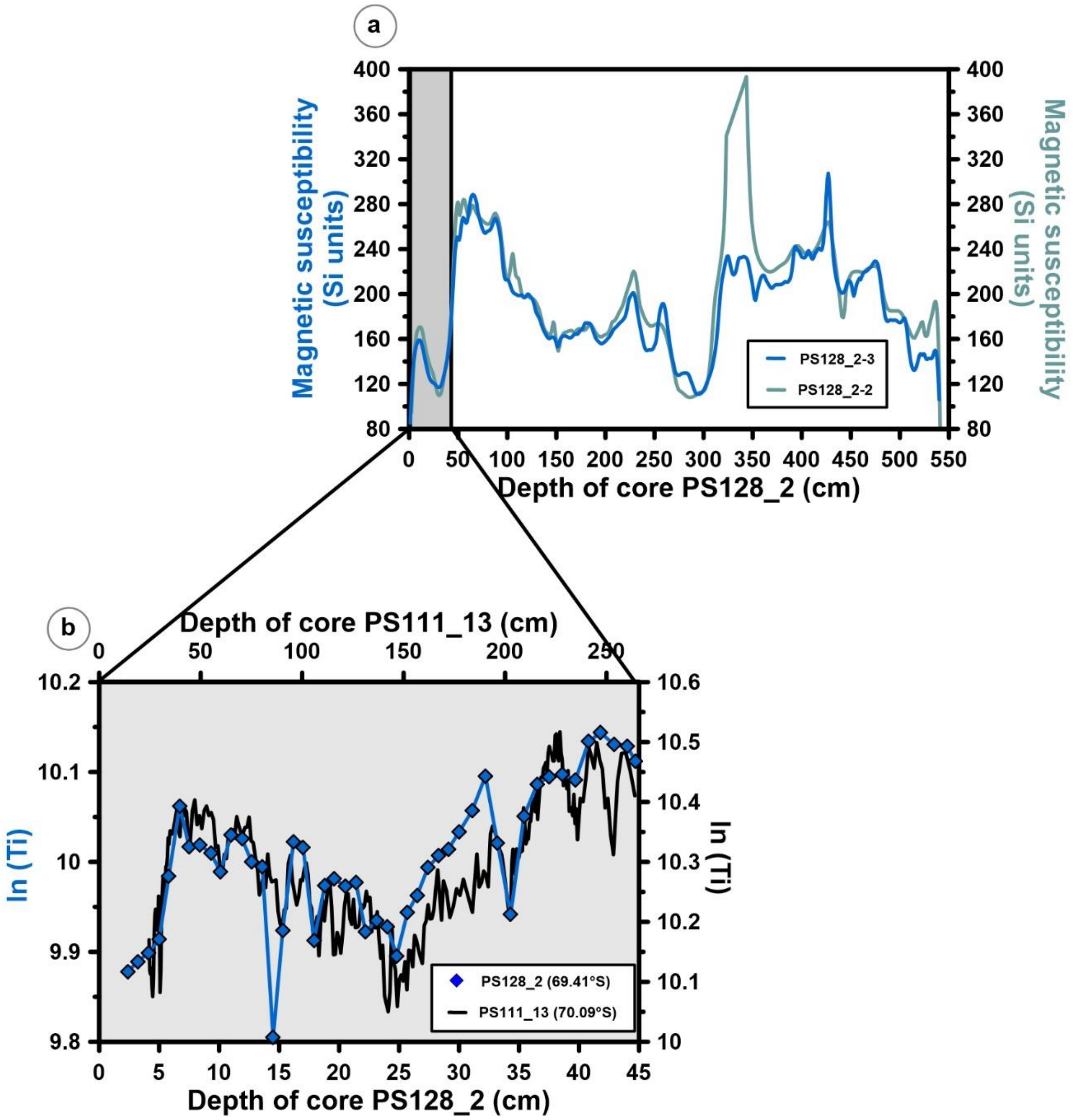

Supplementary Figure 1. Core-to-core correlation. **a,** Magnetic susceptibility correlation between neighboring cores PS128_2-2 (69.4115°S, 5.5897°W) and PS128_2-3 (69.4107°S, 5.5890°W) (<100 m apart from each other). **b,** ln (Ti) correlation between core PS128_2 and PS111_13 (70. 0936°S, 6. 8501°W) for the uppermost 45 cm.

Supplementary Table 1. Calibrated planktonic ^{14}C ages with 1-σ age range of core PS128_2 for the last glacial period and the transferred ages from core PS111_13 for the Holocene period.

AWI nr.	Depth (cm) PS128_2	Depth (cm) PS111_13	AMS ^{14}C age (kyr)	+-(y)	Cal kyr (BP)	ΔR 666 ^{14}C 1-σ age range (kyr)	
10687.1.1 and 10694.1.1	*0.5		1.178	64	0.375	0.375	0.375
4646.1	26.7	157.5	8.260	143	7.858	7.700	8.016
4831.1	28.7	157.5	8.136	107	7.745	7.623	7.866
4832.1	31	170.5	9.682	112	9.546	9.394	9.699
4647.1	31.5	178.5	9.410	95	9.236	9.099	9.373
4835.1	33.8	182.5	9.885	99	9.784	9.612	9.956
4836.1	34.4	194	9.810	111	9.696	9.530	9.861
4648.1	35	198	9.947	102	9.902	9.727	10.077
4837.1	40	202	10.650	102	10.874	10.704	11.044
4839.1	43	254	11.330	107	11.819	11.618	12.019
4840.1	44.5	266	10.998	112	11.334	11.162	11.506
**AWI nr.**	**Depth (cm)**	**Depth (cm) PS111_13**	**AMS ^{14}C age (kyr)**	**+-(y)**	**Cal kry (BP)**	**ΔR 1200 ^{14}C 1-σ age range (kyr)**	
10690.1.1 and 10699.1.1	*79.5		15.284	147	16.192	15.972	16.411
12255.1.1	82.5		15.638	128	16.647	16.455	16.852
12256.1.1	84.5		16.077	141	17.182	16.978	17.370
12257.1.1	86.5		16.262	132	17.415	17.200	17.632
12258.1.1	88.5		17.042	139	18.336	18.171	18.538
12259.1.1	90.5		17.409	150	18.708	18.533	18.914
10701.1.1 and 10692.1.1	*97.5		17.843	174	19.185	18.951	19.406
12263.1.1	98.5		18.318	150	19.762	19.538	19.972
12264.1.1	100.5		18.886	156	20.449	20.241	20.666
12265.1.1	102.5		19.318	161	20.971	20.735	21.213
12267.3.1	106.5		19.494	209	21.202	20.875	21.482
12268.1.1	108.5		19.727	378	21.480	21.011	21.958
11958.1.1 and 11969.1.1	*110.5		20.633	179	22.531	22.323	22.752
12269.1.1	112.5		20.828	173	22.717	22.510	22.920
11970.1.1 and 11959.1.1	*124.5		24.420	232	26.605	26.345	26.898
11971.1.1 and 11960.1.1	*140.5		30.774	380	33.158	32.711	33.743
11961.1.1 and 11972.1.1	*160.5		36.845	643	39.784	39.241	40.400
12222.1.1 and 12223.1.1	**176		41.231	379	42.588	42.381	42.775

* Represents the combination between size fractions 125-250 and 250-500 µm.
** Represents the mean between 171.5 and 180.5 cm sample depth intervals.

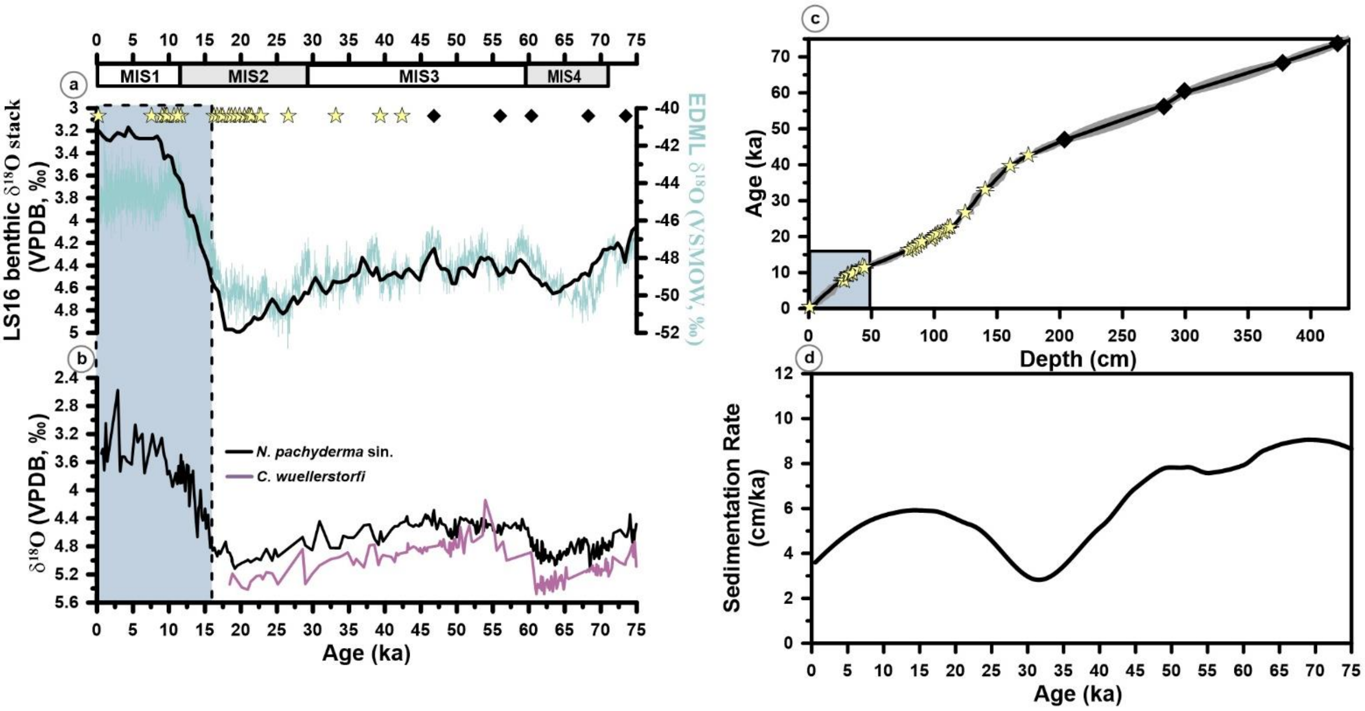

Supplementary Figure 2. Age model of core PS128_2. a, Correlation between LS16 benthic stack [7] and EDML [10] ice core δ^{18}O records. **b,** *Neogloboquadrina pachyderma* sinistral (planktonic) and *Cibicides wuerllerstorfi* (benthic) δ^{18}O records from core PS128_2. We apply a 0.64 ‰ interspecific offset to adjust for seawater disequilibrium in *C. wuellerstorfi* [11]. **c,** Bayesian age-depth model [8] of core PS128_2 within PaleoDataView v. 0.8.3.4 (Ref. [9]). **d,** Sedimentation rate in cm/ka. Yellow stars represent calibrated radiocarbon ages and black triangles depict stratigraphic tie-points based on δ^{18}O alignment. The blue area spanning the last 15 ka indicates the typical [12–14] sedimentary sequence marked by a sharp limitation of biogenic carbonate due to low productivity of foraminiferal shells and their preservation at our site location.

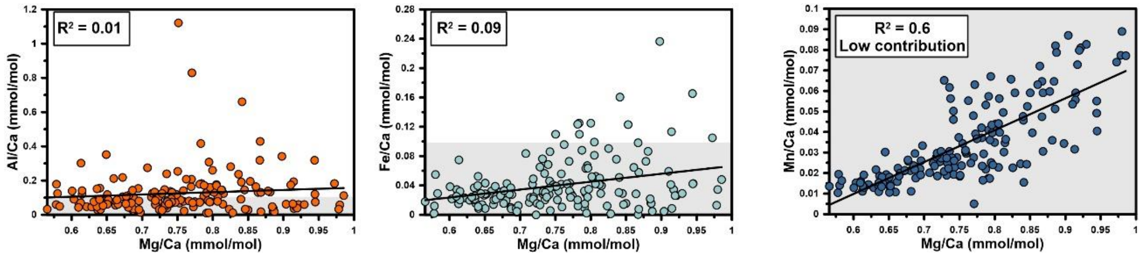

Supplementary Figure 3. Assessment of sample contamination. Panels show the comparison between Mg/Ca with Al/Ca, Fe/Ca, and Mn/Ca. Shading marks the commonly accepted threshold values for contamination of Barker et al. [18].

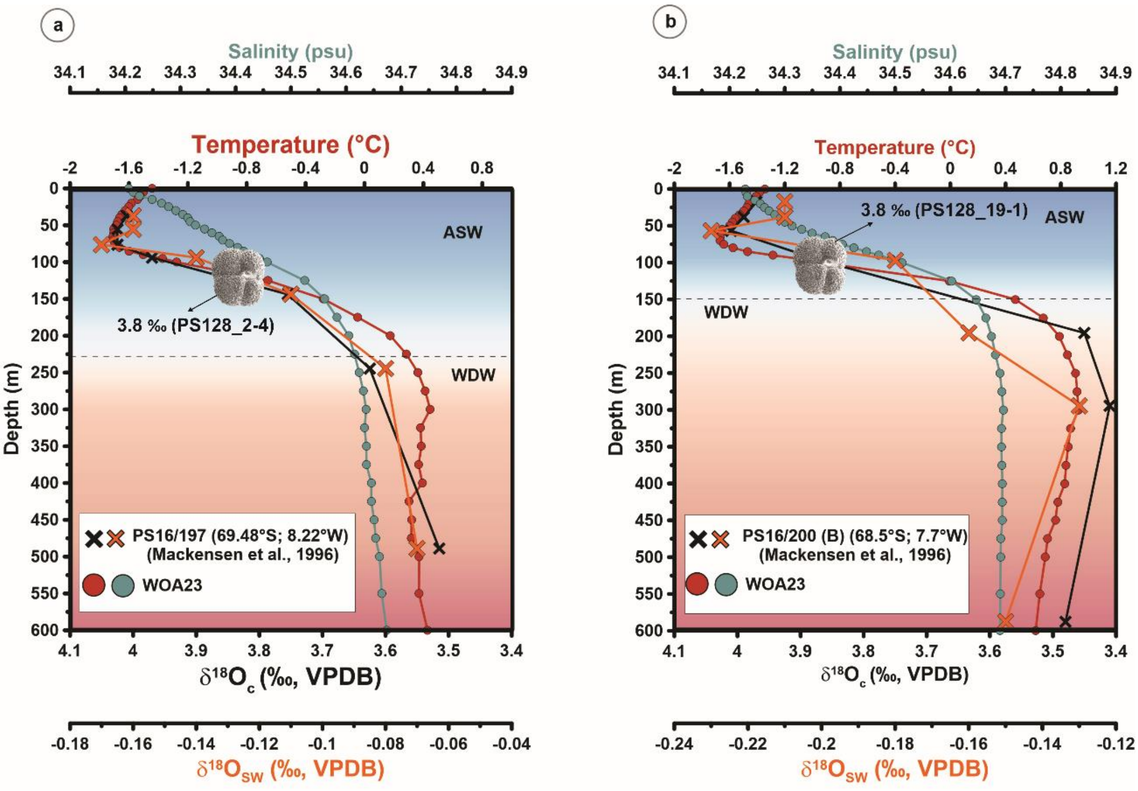

Supplementary Figure 4. Oceanographic profile and apparent calcification depth of *N. pachyderma* sin. in our studied region. Depth profiles showing annual temperature and salinity (WOA 23) [24] compared with seawater δ^{18}O measurements and calculated predicted calcite δ^{18}O values (Mackensen et al. [13]) at two stations (**a**) PS16/197 (69.48°S; 8.22°W) and (**b**) PS16/200 (68.5°S; 7.7°W). White stars represent the apparent calcification depth of *N. pachyderma* sin. at 100 m and 120 m water depth for 68.5°S and 69.48°S, respectively. The apparent calcification depth is based on surface *N. pachyderma* sin. δ^{18}O values from surface MUC cores PS128_2-4 (-69.41°S; 5.6°W) and PS128_19-1 (68.53°S; 7°W).

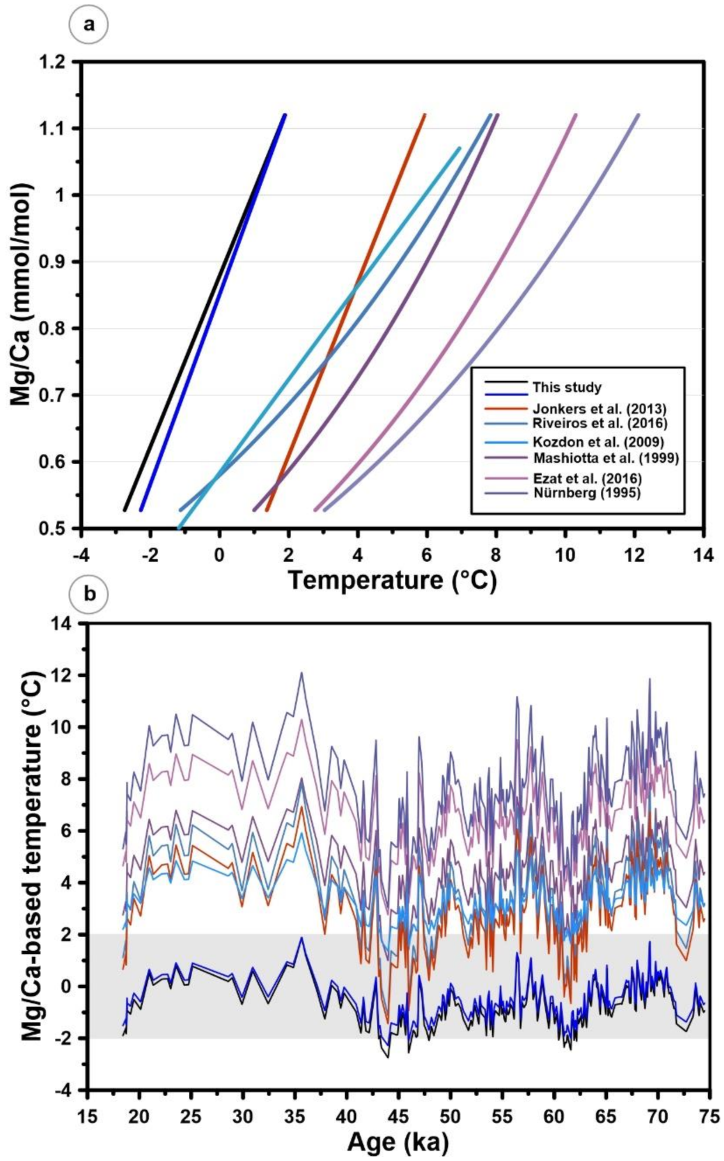

Supplementary Figure 5. Mg/Ca-based subsurface temperature assessment. a, and **b,** Mg/Ca-based temperature based on the available equations (Refs. [29–35]) compared to our temperature calibration (subST$_{Mg/Ca\ vs.\ \Delta47}$). The grey area represents the maximum modern temperature range at our site location varying from +2°C (Warm Deep Water, WDW) to -2°C (Antarctic Surface Water, ASW).

Supplementary Table 2. Summarized temperatures from available Mg/Ca calibration equations for *N. pachyderma* in comparison to our study.

Reference	Maximum Temperature (°C) (1.12 mmol/mol)	Minimum Temperature (°C) (0.53 mmol/mol)	Amplitude (°C)	Average Temperature (°C)
Nürnberg [29]	12.11	3.03	9.07	7.75
Vázquez Riveiros et al. [30]	7.83	-1.13	8.97	3.52
Jonkers et al. [35]	6.94	-1.43	8.37	2.91
Ezat et al. [33]	10.3	2.76	7.53	6.68
Mashiotta et al. [31]	8.04	1	7.04	4.65
Kozdon et al. [32]	5.92	1.36	4.56	3.37
This study (Mg/Ca x Δ_{47})	1.88 ± 0.33	-2.75 ± 0.79	4.63	-0.71
This study (cold/warm end-member)	1.89 ±0.12	-2.28 ±0.19	4.17	-0.45

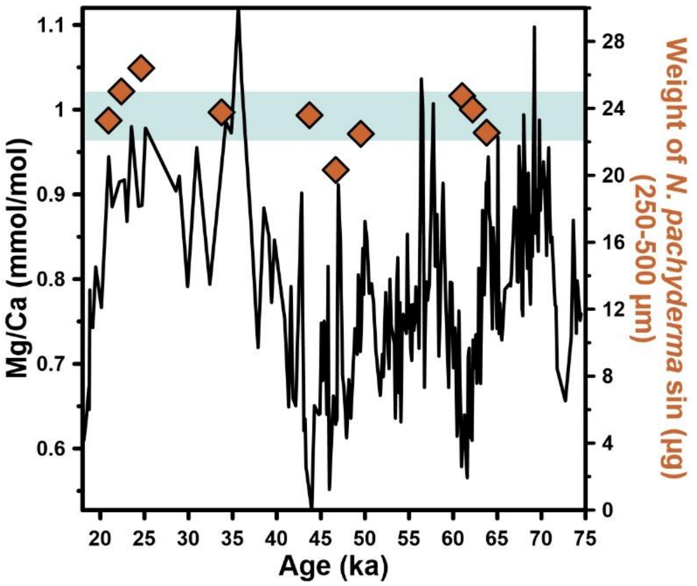

Supplementary Figure 6. Relationship between Mg/Ca ratios and weights of *N. pachyderma* sin. across the last glacial period. Weights averaged from of ~80 individuals of *N. pachyderma* sin. (250-500 µm) (23.60 ± 1.56 µg, light green bar) indicate no carbonate dissolution in relationship to foraminiferal Mg/Ca ratios.

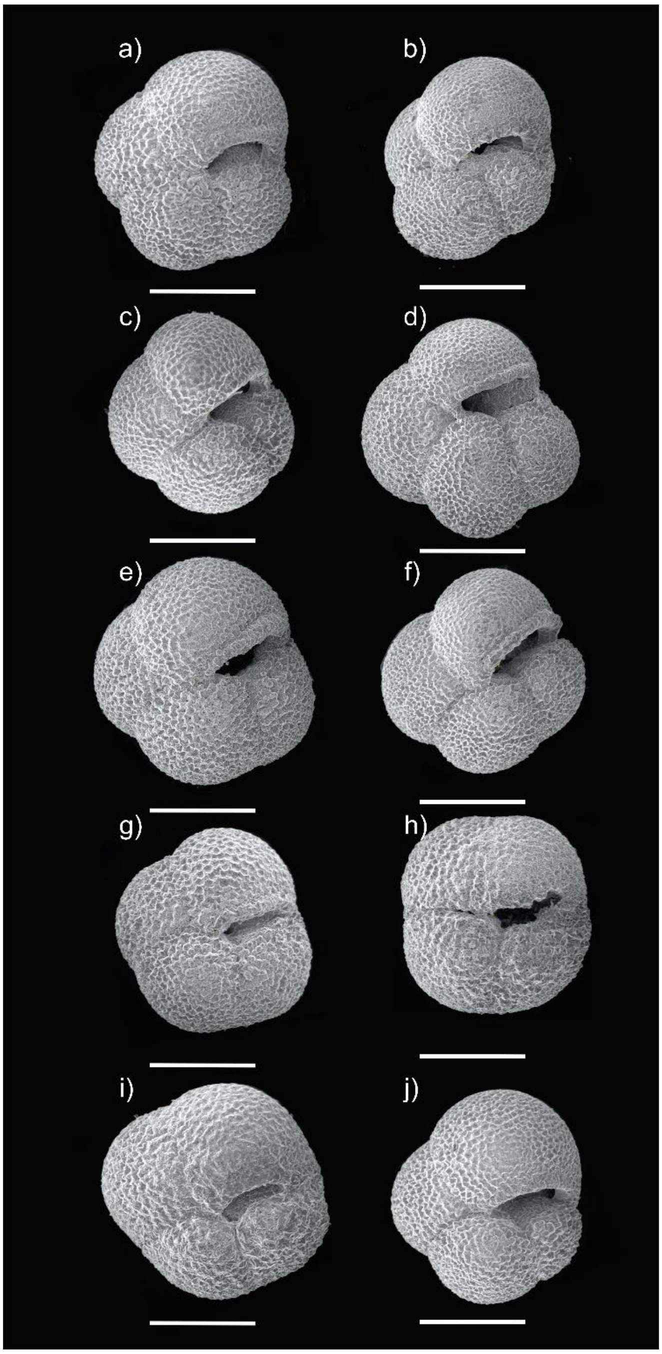

Supplementary Figure 7. Scanning electron microscopy (SEM) images of *Neogloboquadrina pachyderma* sinistral from core PS128_2 during the last glacial period (a: 104.5 cm; b: 112.5 cm; c: 118.5 cm; d: 144.5 cm; e: 179.5 cm; f: 200.5 cm; g: 220.5 cm; h: 311.5 cm; i: 320.5 cm; j: 333.5 cm). The specimens are well-preserved, with no significant signs of dissolution or breakage (e.g., sharp edges of the chambers and sutures as well as the surface texture). White lines represent scale bars (200 µm).

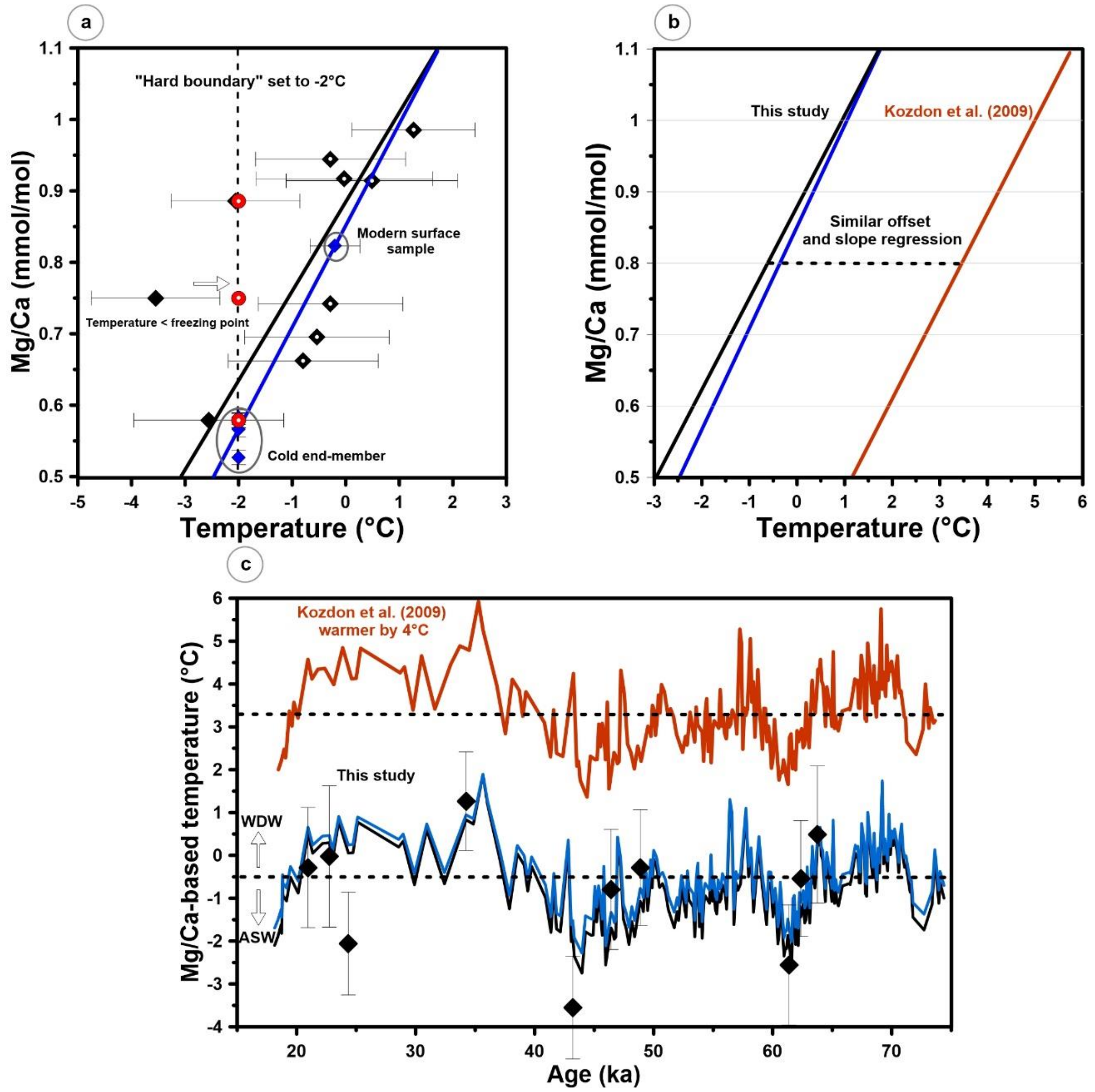

Supplementary Figure 8. Mg/Ca ratio independently calibrated against Δ_{47}-based subsurface temperature in comparison with Kozdon et al. [32]. **a,** Linear relationship between Mg/Ca and Δ_{47} (r=0.75; r^2=0.57) (black line) and between the cold end-member and modern surface Mg/Ca sample (cold-warm end-member; blue line). The deviation between the two linear regressions is small. **b,** Comparison between slopes of our established Mg/Ca vs. Δ_{47}-temperature (subST$_{Mg/Ca\ vs.\ \Delta47}$) (black and blue lines) and Kozdon et al. [32] (orange line). **c,** Mg/Ca-based temperature reconstruction using our approaches (subST$_{Mg/Ca\ vs.\ \Delta47}$ and cold-warm end-member) and Kozdon et al. [32]. The comparison between our linear regression and that of Kozdon et al. [32] presents strongly consistent temperature offset of 4°C, attributable to the close agreement in slope steepness (i.e., slope angle). The error bars show the confidence intervals of Δ_{47} temperature from the Monte Carlo approach (68 % Confidence Interval). Dashed lines represent the average temperature of our approaches and that of Kozdon et al. [32].

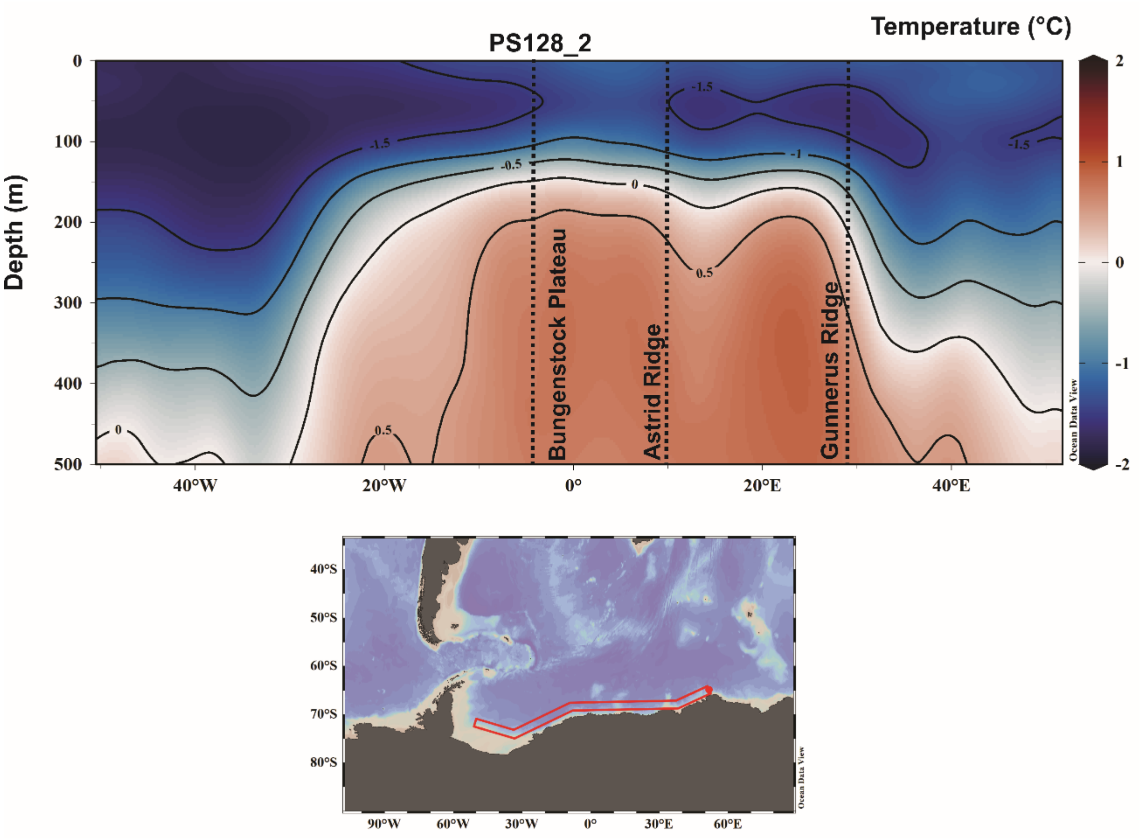

Supplementary Fig. 9. Warm Deep Water updoming from 30°E to 10°W. Temperature profile for the upper 500 m of the water column from a longitudinal transect, which represents the preferential westward WDW transport within the Weddell Gyre. Vertical dashed lines depict the longitudes positions of Gunnerus Ridge, Astrid Ridge and Bungenstock Plateau.

Supplementary References

1. Tiedemann, Ralf and Müller, J. The Expedition PS128 of the Research Vessel POLARSTERN to the Weddell Sea, Lazarew Sea, Riiser-Larsen Sea, Cosmonaut Sea and Cooperation Sea in 2022. *Berichte zur Polar- und Meeresforschung = Reports on polar and marine research, 764*, pp. 1–235 (2022).

2. Schröder, M. The expedition PS111 of the research vessel POLARSTERN to the southern Weddell Sea in 2018. *Berichte zur Polar - und Meeresforschung = Reports on Polar and Marine Research, 718*, pp. 1–161 (2018).

3. Mollenhauer, G., Grotheer, H., Gentz, T., Bonk, E. & Hefter, J. Standard operation procedures and performance of the MICADAS radiocarbon laboratory at Alfred Wegener Institute (AWI), Germany. *Nucl. Instruments Methods Phys. Res. Sect. B Beam Interact. with Mater. Atoms* **496**, 45–51 (2021).

4. Heaton, T. J. *et al.* Marine20—The Marine Radiocarbon Age Calibration Curve (0–55,000 cal BP). *Radiocarbon* **62**, 779–820 (2020).

5. Heaton, T. J. *et al.* MARINE RADIOCARBON CALIBRATION IN POLAR REGIONS: A SIMPLE APPROXIMATE APPROACH USING MARINE20. *Radiocarbon* **65**, 848–875 (2023).

6. Reimer, P. J. & Reimer, R. W. A Marine Reservoir Correction Database and On-Line Interface. *Radiocarbon* **43**, 461–463 (2001).

7. Lisiecki, L. E. & Stern, J. V. Regional and global benthic $\delta^{18}O$ stacks for the last glacial cycle. *Paleoceanography* **31**, 1368–1394 (2016).

8. Blaauw, M. & Christeny, J. A. Flexible paleoclimate age-depth models using an autoregressive gamma process. *Bayesian Anal.* **6**, 457–474 (2011).

9. Langner, M. & Mulitza, S. Technical note: PaleoDataView - A software toolbox for the collection, homogenization and visualization of marine proxy data. *Clim. Past* **15**, 2067–2072 (2019).

10. Barbante, C. *et al.* One-to-one coupling of glacial climate variability in Greenland and Antarctica. *Nature* **444**, 195–198 (2006).

11. Shackleton, N. J. & Opdyke, N. D. Oxygen Isotope and Palaeomagnetic Stratigraphy of Equatorial Pacific Core V28-238: Oxygen Isotope Temperatures and Ice Volumes on a 10^5 Year and 10^6 Year Scale. *Quat. Res.* **3**, 39–55 (1973).

12. Mackensen, A., Grobe, H., Hubberten, H.-W., Spiess, V. & Fütterer, D. . Stable isotope stratigraphy from the Antarctic continental margin during the last one million years. *Mar. Geol.* **87**, 315–321 (1989).

13. Mackensen, A., Hubberten, H. ‐ W., Scheele, N. & Schlitzer, R. Decoupling of $\delta^{13}C_{\Sigma CO2}$ and phosphate in recent Weddell Sea deep and bottom water: Implications for glacial Southern Ocean paleoceanography. *Paleoceanography* **11**, 203–215 (1996).

14. Smith, J. A., Hillenbrand, C.-D., Pudsey, C. J., Allen, C. S. & Graham, A. G. C. The presence of polynyas in the Weddell Sea during the Last Glacial Period with implications for the reconstruction of sea-ice limits and ice sheet history. *Earth Planet. Sci. Lett.* **296**, 287–298 (2010).

15. Boyle, E. A. & Keigwin, L. D. Comparison of Atlantic and Pacific paleochemical records for the last 215,000 years: changes in deep ocean circulation and chemical inventories. *Earth Planet. Sci. Lett.* **76**, 135–150 (1985).

16. Boyle, E. A. & Rosenthal, Y. Chemical hydrography of the South Atlantic during the Last Glacial Maximum: Cd vs. d13C: *The South Atlantic: Present and Past Circulation*, edited by G. Wefer et al., pp. 423 – 443, Springer-Verlag, New York (1996).

17. Greaves, M. *et al.* Interlaboratory comparison study of calibration standards for foraminiferal Mg/Ca thermometry. *Geochemistry, Geophys. Geosystems* **9**, (2008).

18. Barker, S., Greaves, M. & Elderfield, H. A study of cleaning procedures used for foraminiferal Mg/Ca paleothermometry. *Geochemistry, Geophys. Geosystems* **4**, 1–20 (2003).

19. Grant, K. M. *et al.* Rapid coupling between ice volume and polar temperature over the past 150,000 years. *Nature* **491**, 744–747 (2012).

20. Shackleton, N. J. Attainment of isotopic equilibrium between ocean water and the benthonic foraminifera genus Uvigerina: isotopic changes in the ocean during the last glacial. (1974).

21. Hut, G. Consultants' Group Meeting on Stable Isotope Reference Samples for Geochemical and Hydrological Investigations, Rep. to Dir. Gen., Vienna, 16–18 September 1985, Int. At. Energy Agency, Vienna, p. 428 (1987).

22. Riethdorf, J., Max, L., Nürnberg, D., Lembke ‐ Jene, L. & Tiedemann, R. Deglacial development of (sub) sea surface temperature and salinity in the subarctic northwest Pacific: Implications for upper ‐ ocean stratification. *Paleoceanography* **28**, 91–104 (2013).

23. Caley, T. & Roche, D. M. Modeling water isotopologues during the last glacial: Implications for quantitative paleosalinity reconstruction. *Paleoceanography* **30**, 739–750 (2015).

24. R.A. Locarnini, A.V. Mishonov, O.K. Baranova, J.R. Reagan, T.P. Boyer, D. Seidov, Z. W. & H.E. Garcia, C. Bouchard, S.L. Cross, C.R. Paver, and D. D. World Ocean Atlas 2023, Volume 1: Temperature. *NOAA Atlas NESDIS* **89**, 52 (2024).

25. Meinicke, N., Reimi, M. A., Ravelo, A. C. & Meckler, A. N. Coupled Mg/Ca and Clumped Isotope Measurements Indicate Lack of Substantial Mixed Layer Cooling in the Western Pacific Warm Pool During the Last ~5 Million Years. *Paleoceanogr. Paleoclimatology* **36**, (2021).

26. Bernasconi, S. M. *et al.* InterCarb: A Community Effort to Improve Interlaboratory Standardization of the Carbonate Clumped Isotope Thermometer Using Carbonate Standards. *Geochemistry, Geophys. Geosystems* **22**, (2021).

27. Daëron, M. & Gray, W. R. Revisiting Oxygen ‐ 18 and Clumped Isotopes in Planktic and Benthic Foraminifera. *Paleoceanogr. Paleoclimatology* **38**, (2023).

28. Daëron, M. & Vermeesch, P. Omnivariant Generalized Least Squares regression: Theory, geochronological applications, and making the case for reconciled $\Delta 47$ calibrations. *Chem. Geol.* **647**, 121881 (2024).

29. Nürnberg, D., Bijma, J. & Hemleben, C. Assessing the reliability of magnesium in foraminiferal calcite as a proxy for water mass temperatures. *Geochim. Cosmochim. Acta* **60**, 803–814 (1996).

30. Vázquez Riveiros, N. *et al.* Mg/Ca thermometry in planktic foraminifera: Improving paleotemperature estimations for *G. bulloides* and *N. pachyderma* left. *Geochemistry, Geophys. Geosystems* **17**, 1249–1264 (2016).

31. Mashiotta, T. A., Lea, D. W. & Spero, H. J. Glacial–interglacial changes in Subantarctic sea surface temperature and $\delta^{18}O$-water using foraminiferal Mg. *Earth Planet. Sci. Lett.* **170**, 417–432 (1999).

32. Kozdon, R., Eisenhauer, A., Weinelt, M., Meland, M. Y. & Nürnberg, D. Reassessing Mg/Ca temperature calibrations of Neogloboquadrina pachyderma (sinistral) using paired $\delta^{44/40}Ca$ and Mg/Ca measurements. *Geochemistry, Geophys. Geosystems* **10**, (2009).

33. Ezat, M. M., Rasmussen, T. L. & Groeneveld, J. Reconstruction of hydrographic changes in the southern Norwegian Sea during the past 135 kyr and the impact of different foraminiferal Mg/Ca cleaning protocols. *Geochemistry, Geophys. Geosystems* **17**, 3420–3436 (2016).

34. Livsey, C. M. *et al.* High ‐ Resolution Mg/Ca and $\delta^{18}O$ Patterns in Modern Neogloboquadrina pachyderma From the Fram Strait and Irminger Sea. *Paleoceanogr. Paleoclimatology* **35**, (2020).

35. Jonkers, L., Jiménez-Amat, P., Mortyn, P. G. & Brummer, G.-J. A. Seasonal Mg/Ca variability of N. pachyderma (s) and G. bulloides: Implications for seawater temperature reconstruction. *Earth Planet. Sci. Lett.* **376**, 137–144 (2013).

36. Regenberg, M. *et al.* Assessing the effect of dissolution on planktonic foraminiferal Mg/Ca ratios: Evidence from Caribbean core tops. *Geochemistry, Geophys. Geosystems* **7**, (2006).

37. Locarnini, R.A., A.V. Mishonov, O.K. Baranova, T.P. Boyer, M.M. Zweng, H.E. Garcia, J.R. Reagan, D. Seidov, K.W. Weathers, C.R. Paver, and I. V. S. (2019). World Ocean Atlas 2018 , Volume 1 : Temperature NOAA Atlas NESDIS 81 WORLD OCEAN ATLAS 2018 Volume 1 : Temperature National Oceanic and Atmospheric Administration. *World Ocean Atlas 2018, Vol. 1 Temp. A. Mishonov, Tech. Ed. NOAA Atlas NESDIS 81, 52pp.* **1**, 52 (2019).
